# Relationship Between Physical Literacy and Cardiorespiratory Fitness in Children and Adolescents: A Systematic Review and Meta-analysis

**DOI:** 10.1007/s40279-024-02129-7

**Published:** 2024-11-23

**Authors:** Tianle Jiang, Guanggao Zhao, Jinmei Fu, Shunli Sun, Ruiming Chen, Delong Chen, Xuewen Hu, Yunong Li, Fanchao Shen, Jin Hong, Haihua Hu

**Affiliations:** 1 School of Physical Education, Nanchang University, Nanchang, 330031 China; 2 Jiangxi Sports Science Medicine Center, Nanchang, 330006 China

## Abstract

**Background:**

Physical literacy (PL) can positively affect the health of children, adolescents, and adults, and is closely related to cardiorespiratory fitness (CRF).

**Objective:**

To perform a systematic review and meta-analysis to examine the relationship between overall physical literacy (PL) and CRF in children and adolescents.

**Methods:**

Cross-sectional, cohort and experimental studies on the relationship between PL and CRF in children and adolescents were collected by searching the Web of Science Core Collection, PubMed, EBSCOhost, ScienceDirect, Cochrane Library and China National Knowledge Infrastructure (CNKI) databases. Based on the characteristics of the included literature, PL was divided into physical competence (PC), daily behavior (DB), knowledge and understanding (K&U), and motivation and confidence (M&C). R 4.3.6 was used to analyze the combined effect size of PL and the relationships of the four dimensions with CRF among children and adolescents.

**Results:**

A total of 21 articles were included, 42.9% of which were published after 2020, involving a total of 43,352 children and adolescents from 23 countries or regions. The characteristics of the included studies showed that, except for the K&U of children and adolescents aged 13–18 years, the K&U of other age groups and the PL, PC, DB, M&C of all age groups were significantly positively correlated with CRF. Furthermore, the results of male and female samples in all included studies were the same. The results of the meta-analysis indicated that PL (COR = 0.64, 95% CI 0.58, 0.70), PC (COR = 0.74, 95% CI 0.69, 0.79), DB (COR = 0.49, 95% CI 0.40, 0.57), K&U (COR = 0.41, 95% CI 0.23, 0.56), and M&C (COR = 0.45, 95% CI 0.41, 0.49) were significantly positively correlated with CRF. Regarding DB, total physical activity (TPA) was positively correlated with CRF (COR = 0.49, 95% CI = 0.40, 0.57). Moderate to vigorous physical activity (MVPA), vigorous physical activity (VPA) and high physical activity (HPA) were positively correlated with CRF (COR = 0.16, 95% CI 0.09, 0.22; COR = 0.33, 95% CI 0.22, 0.43; COR = 0.38, 95% CI 0.13, 0.58), but light physical activity (LPA) was negatively correlated with CRF (COR = − 0.20, 95% CI − 0.32, 0.06).

**Conclusion:**

PL and CRF are positively correlated among children and adolescents, suggesting that the development of physical literacy has a wide range of effects on children and adolescents’ cardiopulmonary health and that these effects are not limited by PL and its various dimensions or sex. In addition, to exert the positive effect of daily activities on cardiopulmonary health, the intensity of physical activity should reach a moderate level or above.

## Key Points

**Table:** 

Physical literacy can improve the cardiorespiratory fitness level of children and adolescents and can promote the comprehensive and healthy development of children and adolescents.
Physical competence, daily behavior (DB), knowledge and understanding, motivation and confidence all showed significant positive correlations with cardiorespiratory fitness.
Physical literacy and DB can promote cardiorespiratory fitness in children and adolescents.

## Introduction

Cardiorespiratory fitness (CRF) refers to the body’s ability to take in, transport, and utilize oxygen [1]. Research has confirmed that CRF is closely associated with chronic cardiovascular diseases and high cholesterol levels; furthermore, CRF has become an important predictor of mortality [2]. For children and adolescents, CRF is not only an important indicator of overall health but also a critical factor affecting their future health outcomes [3]. However, the current CRF of children and adolescents is under serious threat. Research by the American Heart Association (AHA) revealed that nearly 60% of children and adolescents have poor CRF [3]. In China, CRF is declining among children and adolescents, with as many as 66–85% of adolescents having below-normal CRF levels [4]. Therefore, exploring warning indicators and intervention strategies for CRF in children and adolescents is highly important for the development of a healthy society both in China and globally. Studies have shown that developing PL early in life promotes physical health in children while affecting PA participation and health levels in adulthood. In addition, PL is not equal to PA, and PA cannot be used as a substitute for PL. PL and PA can be mutually reinforcing. PL does not have to be manifested in PA; people who are unable to participate in PA can also benefit from PL because PL includes not only sports but also theoretical knowledge [5].

Studies have shown that promoting physical literacy (PL) can have a positive impact on the health of children, adolescents, and adults [6, 7]. As an important component of health assessments, CRF is influenced by various factors, such as lifestyle, physical activity, cognitive abilities, and mental health [8]. PL encompasses four dimensions: physical competence (PC), daily behavior (DB), knowledge and understanding (K&U), and motivation and confidence (M&C) [9, 10]. Each dimension is closely related to factors influencing CRF, suggesting that PL could be a potential warning indicator and intervention direction for CRF.

In recent years, especially since 2020, there has been a growing number of studies exploring the relationship between PL and CRF among children and adolescents. However, this research is in the early stage, and thus, the results are fragmented. Investigators often conduct empirical research on the relationship between overall PL or a specific dimension and CRF based on their individual research interests and environmental conditions. Currently, there are no systematic reviews or meta-analyses of studies examining the relationship between PL and CRF among children and adolescents. Therefore, this study aimed to provide a scientific basis for establishing indicators for early warning and intervention strategies for CRF in children and adolescents by systematically reviewing and meta-analyzing published research findings.

## Research Methods

### Literature Search

Our research program has been registered on PROSPERO, the International Prospective Register of Systematic Reviews; Registration number: CRD42023454952.

This study was performed in accordance with the Preferred Reporting Items for Systematic Reviews and Meta-Analyses (PRISMA) guidelines [11]. The Web of Science Core Collection, PubMed, EBSCOhost, ScienceDirect, Cochrane Library, and China National Knowledge Infrastructure (CNKI) electronic databases were searched up to 6 January 2024 to retrieve publicly available studies that examined the relationship between PL and CRF among children and adolescents. The following search terms were used: (1) physical literacy (PL), physical activity, exercise, sports activity, physical competence, motivation and confidence, knowledge and understanding; (2) cardiorespiratory fitness (CRF); and (3) youth, adolescent, child, student, teen. Retrieved articles were screened based on titles and abstracts, followed by full-text evaluation. Additionally, the references of the included studies and relevant research by experts in the field were manually searched to supplement the electronic database search.

### Study Selection

The inclusion criteria were as follows: (1) peer-reviewed English or Chinese journal articles (core journals); (2) studies involving children and adolescents aged 5–18 years; (3) cross-sectional, cohort, or experimental studies; (4) studies related to PL; and (5) studies related to CRF.

The exclusion criteria were as follows: (1) non-English or non-Chinese literature; (2) studies involving subjects with chronic diseases or special populations; and (3) reviews, commentaries, case reports, conference abstracts, etc.

### Data Extraction and Quality Assessment

Two researchers independently extracted the data and assessed the quality of the included studies. In cases of disagreement, a third person summarized the discrepancies, and the research group discussed the disagreements to reach a consensus. The following data were extracted from the included studies: the first author’s name, publication year, characteristics of the study population (sample size, age range, country or region), measurement methods for PL and CRF, outcome measures, correlation coefficients (r-values) or relevant data for calculating correlations between PL and CRF.

Since the included studies were all observational, the Agency for Healthcare Research and Quality (AHRQ) Cross-Sectional Study Quality Assessment Criteria were used for quality assessment [12]. These criteria are used to evaluate studies based on 11 aspects, including data sources, inclusion criteria, observation time, and subjective factors of evaluators. Each study is scored as “yes” (1 point), “no” (0 points), or “unclear” (0 points), with a maximum score of 11 points. Scores ≥ 8 indicate high quality, scores from 4–7 indicate moderate quality, and scores ≤ 3 indicate low quality [13].

### Statistical Analysis

The meta-analysis was performed using R (version 4.3.6). The correlation coefficient (r value) was chosen as the effect size to combine the results [14]. Before conducting the meta-analysis, heterogeneity was assessed using the *I*^2^ test. *I*^2^ quantifies the degree of heterogeneity, ranging from 0 to 100%. If *I*^2^ was ≤ 50%, indicating low heterogeneity, a fixed effects model (common effects model, CEM) was used for the meta-analysis. If* I*^2^ was  > 50%, indicating significant heterogeneity, a random effects model (randomized effects model (REM)) was used for meta-analysis [15]. Sensitivity analysis was also conducted to explore sources of heterogeneity [16].

## Results

### Literature Search Process and Results

**Fig. 1:**
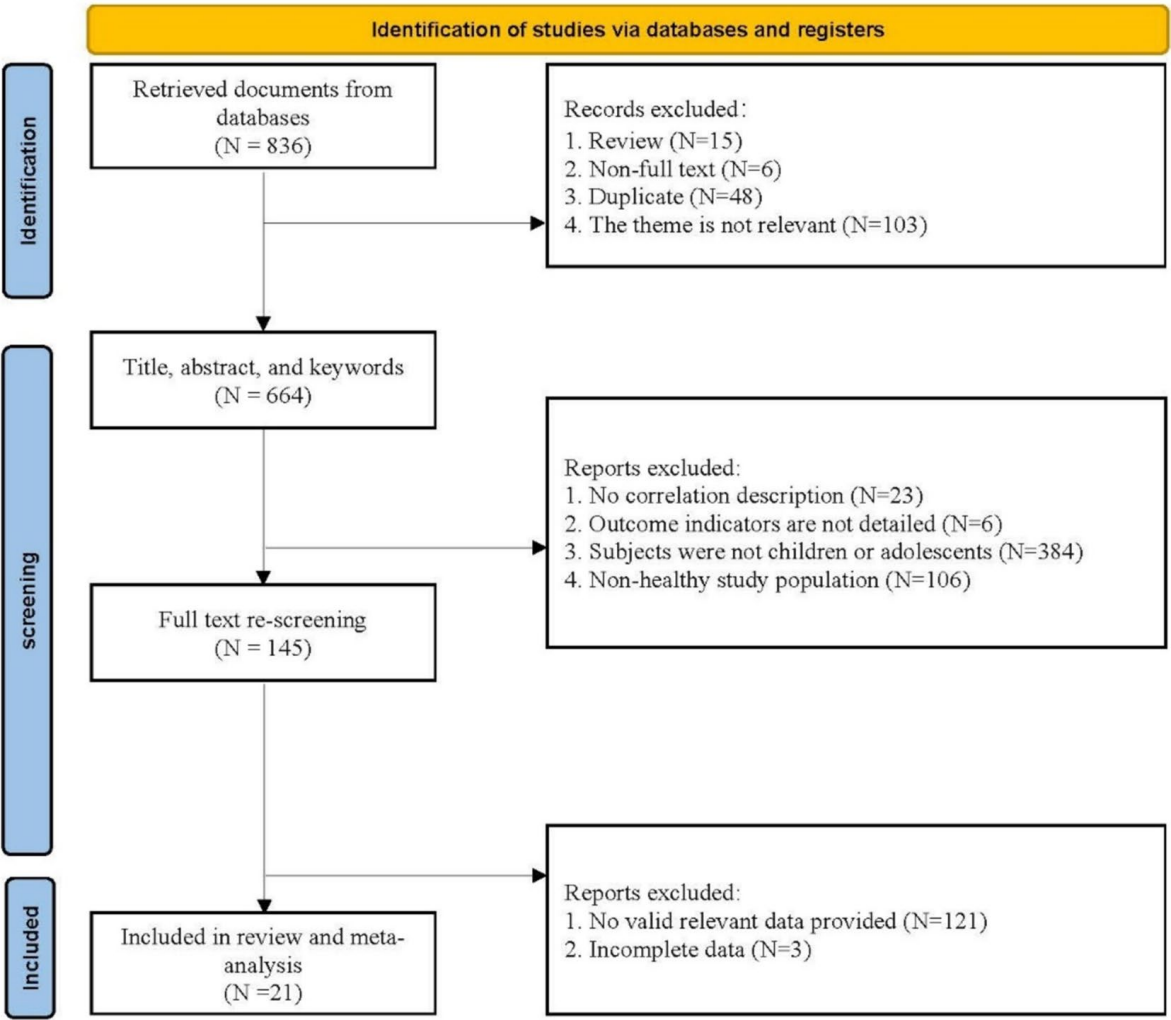
Literature screening process

A total of 836 relevant articles were retrieved. After applying the inclusion and exclusion criteria, 21 articles were ultimately included. The screening process is illustrated in Fig. 1.

### Characteristics of Included Studies and Quality Assessment

A total of 21 articles comprising 27 studies were included (Table 1). All were observational studies, including 20 cross-sectional studies and one cohort study, with no experimental studies. Among them, 85.7% (18 articles) were published after 2016, and 42.9% (nine articles) were published after 2020. The studies involved a total of 43,973 participants from 23 countries or regions, including four studies from Canada, five from mainland China, Hong Kong, and Taiwan, three from the USA, two from the UK, and one each from Spain, Ireland, Greece, Sweden, Finland, Slovenia, Brazil, South Africa, Tanzania, Cote d’Ivoire, Japan, Malaysia, South Korea, Singapore, Thailand, Myanmar, and Bosnia and Herzegovina. Studies with sample sizes exceeding 200, 500, and 1000 participants accounted for 80.9%, 57.1%, and 28.5% of the total, respectively. Various instruments or questionnaires were used to measure PL and CRF. The Canadian Assessment of Physical Literacy (CAPL) was used to assess PC, accelerometers or questionnaires were used to assess DB, and questionnaires were used to assess K&U and M&C. CRF was assessed primarily using the shuttle run test (SRT) or progressive aerobic cardiovascular endurance run (PACER).

**Table 1:** Characteristics of studies included in the review and meta-analysis

Study	Region	Study type	Sample size Female/male	Age, y	PL		CRF	
					Type	Method	Type	Method
Ryu et al. 2021 [17]	USA	Control	134/127	8.27 ± 0.7	DB	Acc	*V*O_2_max	PACER
Gerber et al. 2021 [18]	South Africa	Cross-sectional	626/634	8.3 ± 1.4	DB	Acc	*V*O_2_max	SRT
	Côte d'Ivoire		244/255	8.0 ± 1.6				
	Tanzania		302/291	9.4 ± 1.7				
Jurak et al. 2021 [19]	Slovenia	Cross-sectional	380/333	12–15	DB	Q	*V*O_2_max	SRT
Nevill et al. 2020 [20]	UK	Cross-sectional	3775/4227	10–16	DB	Q	*V*O_2_max	SRT
Kaioglou et al. 2020 [21]	Greece	Cross-sectional	351/364	10.2 ± 1.3	PC, DB, K&U, M&C, PL	CAPL	*V*O_2_max	PACER
Sun et al. 2020 [22]	China	Cross-sectional	536	7–18	DB	Acc	*V*O_2_max	SRT
Calahorro et al. 2020 [23]	Spain	Cross-sectional	60	8–19	DB	Acc	*V*O_2_peak	PV
Gu et al.2020 [24]	US	Cross-sectional	182/192	9.64 ± 1.16	DB	Acc	*V*O_2_max	PACER
Caldwell et al. 2020 [25]	Canada	Cross-sectional	113/109	10.7 ± 1.0	DB, PL	PLAY	HR	Bruce
Kidokoro et al. 2019 [26]	China, Hong Kong, Taiwan, South Korea, Thailand, Myanmar, Singapore, Japan, Malaysia	Cross-sectional	9553	12–15	DB	Q	*V*O_2_max	PACER
Tremblay et al. 2018 [27]	Canada	Cross-sectional	5030/5004	8–12	PC, DB, K&U, M&C, PL	CAPL	*V*O_2_max	PACER
Lang et al. 2018 [28]	Canada	Cross-sectional	266/256	8	PC、DB, K&U, M&C, PL	CAPL	*V*O_2_max	PACER
			477/469	9				
			588/613	10				
			756/754	11				
			274/257	12				
Leppänen et al. 2018 [29]	Sweden	Cross-sectional	40	5.5 ± 0.2	DB	Acc	*V*O_2_max	SRT
Pojskic et al. 2018 [30]	Bosnia & Herzegovina	Cross-sectional	753	10–14	DB	Q	*V*O_2_max	PACER
Coledam et al. 2018 [31]	Brazil	Cross-sectional	344/337	10–17	DB	Q	*V*O_2_max	SRT
Collings et al. 2017 [32]	Finland	Cross-sectional	410	7.6 ± 0.4	DB	Sensor	HR	PM
Comeau et al. 2017 [33]	Canada	Cross-sectional	145	9–12	PC	PLAY	*V*O_2_peak	SRT
Bai et al. 2016 [34]	US	Cross-sectional	692	6–11	DB	Q	*V*O_2_max	PACER
			422	12–15				
Hsieh et al. 2014 [35]	Taiwan	Cross-sectional	1389/1490	12	DB	Q	Time	800m
Denton et al. 2013 [36]	UK	Cross-sectional	81/54	12 ± 1	DB	Acc	*V*O_2_max	PM
Hussey et al. 2007 [37]	Ireland	Cross-sectional	284	7–10	DB	Acc	*V*O_2_max	SRT

*PC* physical competence, *DB* daily behavior, *M&C* motivation and confidence, *K&U* knowledge and understanding, *PL* physical literacy, *Acc* accelerometer, *Q* questionnaire, *CAPL* Canadian Assessment of Physical Literacy, *PLAY* Physical Literacy Assessment in Youth, *VO*_*2*_*max* maximum oxygen uptake, *VO*_*2*_*peak* peak oxygen uptake, *HR* heart rate, *SRT* shuttle run test, *PACER* progressive aerobic capacity endurance run, *PM* power meter, *PV* portable ventilator, *m* meter

**Fig. 2:**
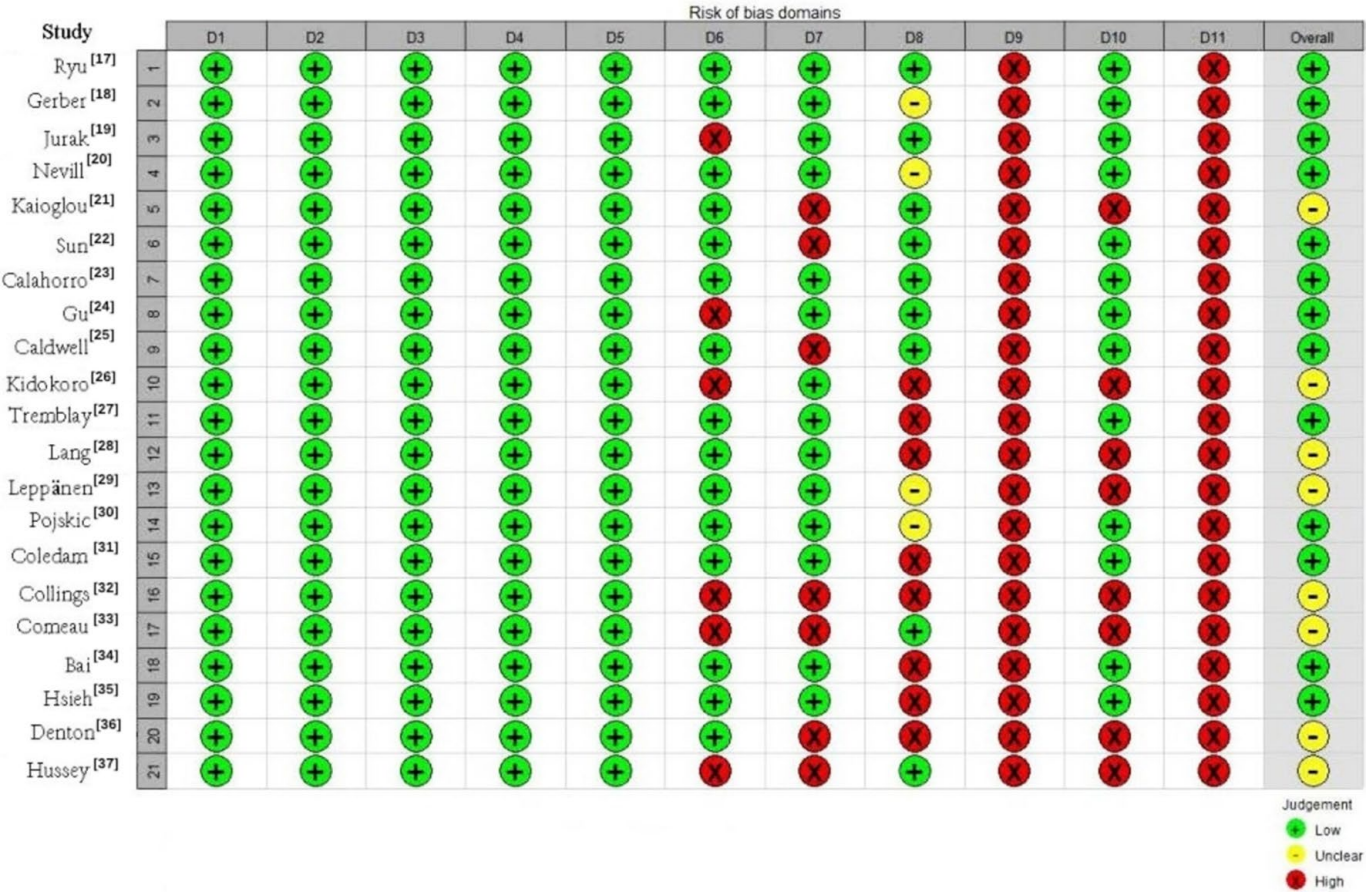
Risk of bias graph of included studies. D1: Defines the source of information (survey, literature review). D2: Lists inclusion and exclusion criteria of exposed and unexposed subjects (case and control) or refer to previous. D3: Indicates time period used for identifying patients. D4: Indicates whether or not subjects were consecutive if not population-based. D5: Indicates if evaluators of subjective components of study were masked to other aspects of the status of the participants. D6: Describes any assessments undertaken for quality assurance purposes (e.g., test/retest of primary outcome measurements). D7: Explains any patient exclusions from analysis. D8: Describes how confounding was assessed and/or controlled. D9: If applicable, explains how missing data were handled in the analysis. D10: Summarizes patient response rate and completeness of data collection. D11: Clarifies what follow-up was expected and the percentage of patients for which incomplete data or follow-up were obtained

The quality assessment results indicated that 13 articles were of high quality, eight were of moderate quality, and no studies were of low quality (Fig. 2).

### Systematic Review and Meta-analysis Results

#### Relationship Between Physical Literacy (PL) and Cardiorespiratory Fitness (CRF) Among Children and Adolescents

**Fig. 3:**
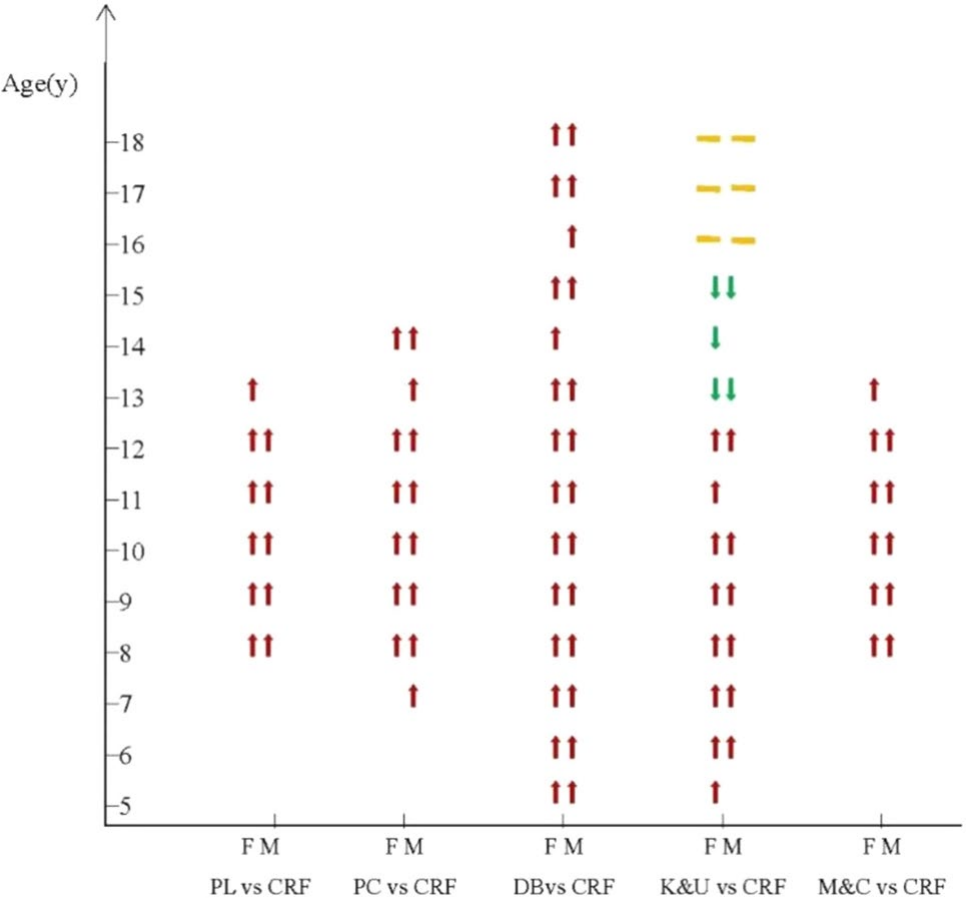
The relationships between PL, its dimensions, CRF and age. *F* female, *M* male, *PL* physical literacy, *PC* physical competence, *DB* daily behavior, taking moderate to vigorous physical activity as an example, *K&U* knowledge and understanding, *M&C* motivation and confidence, *CRF* cardiorespiratory fitness, ⬆ significant positive correlation, ⬇ significant negative correlation, – no significant relationship

**Fig. 4:**
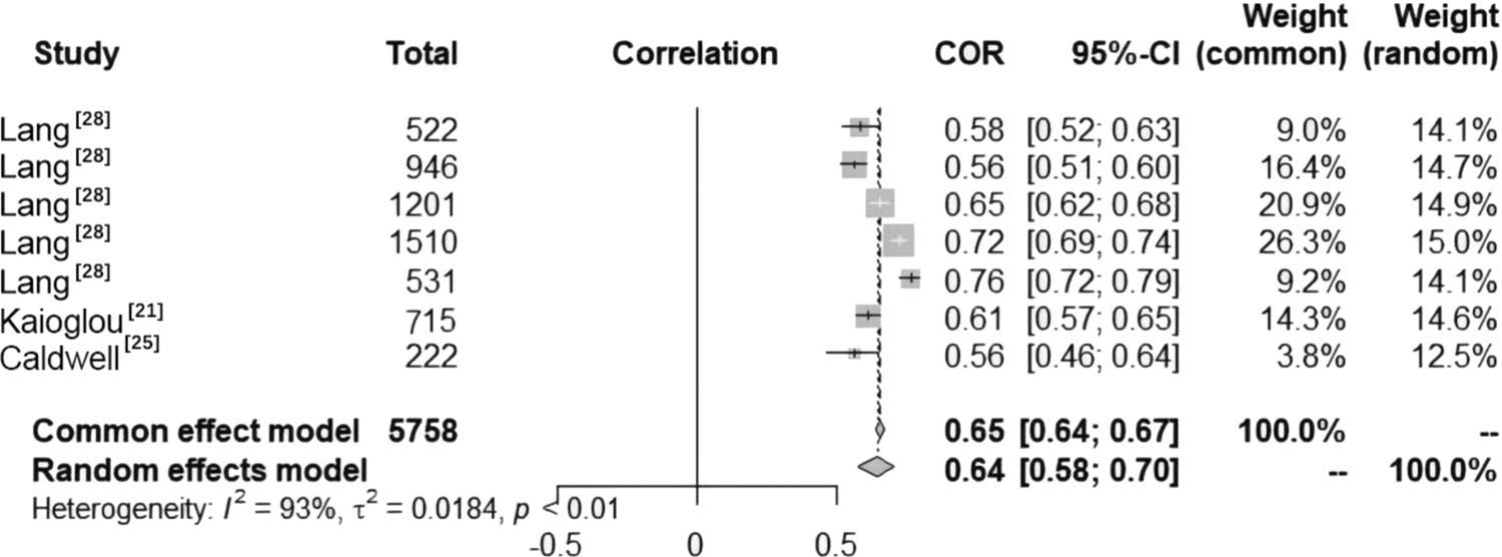
Forest diagram of the correlation between physical literacy (PL) and cardiorespiratory fitness (CRF) in children and adolescents. *COR* correlation

The included studies indicated a significant positive relationship between PL and CRF among children and adolescents aged 8–13 years (Fig. 3). The results were the same for both males and females across different studies. No studies were found for other age groups. Data on the relationships between PL and CRF among children and adolescents were reported in seven studies (Fig. 4). Heterogeneity testing revealed significant heterogeneity among the studies (*I*^2^ > 50%, *P* < 0.01); thus, the random effects model was chosen for the meta-analysis. The meta-analysis revealed a significant positive correlation between PL and CRF among children and adolescents (COR = 0.64, 95% CI 0.58, 0.70).

#### Relationship Between PC and CRF among Children and Adolescents

**Fig. 5:**
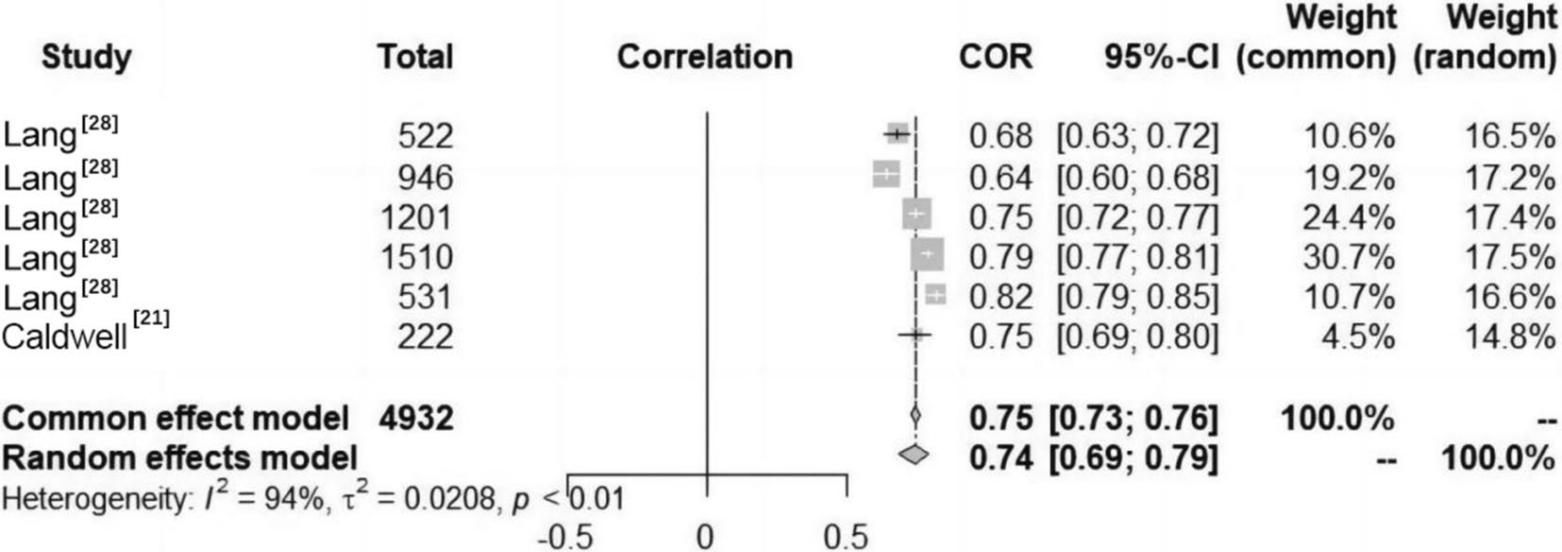
Forest diagram of the correlation between physical competence (PC) and cardiorespiratory fitness (CRF) in children and adolescents. *COR* correlation

The included studies indicated a significant positive correlation between PC and CRF in children and adolescents aged 7–14 years (Fig. 3). The results were the same for both males and females across different studies. No studies examined other age groups. Data on the relationships between PC and CRF among children and adolescents were reported in six studies (Fig. 5). Heterogeneity testing revealed significant heterogeneity among the studies (*I*^2^ > 50%, *P* < 0.01); thus, the random effects model was chosen for the meta-analysis. The meta-analysis revealed a significant positive correlation between PC and CRF in children and adolescents (COR = 0.74, 95% CI 0.69, 0.79).

#### Relationship Between Daily Behavior (DB) and CRF Among Children and Adolescents

**Fig. 6:**
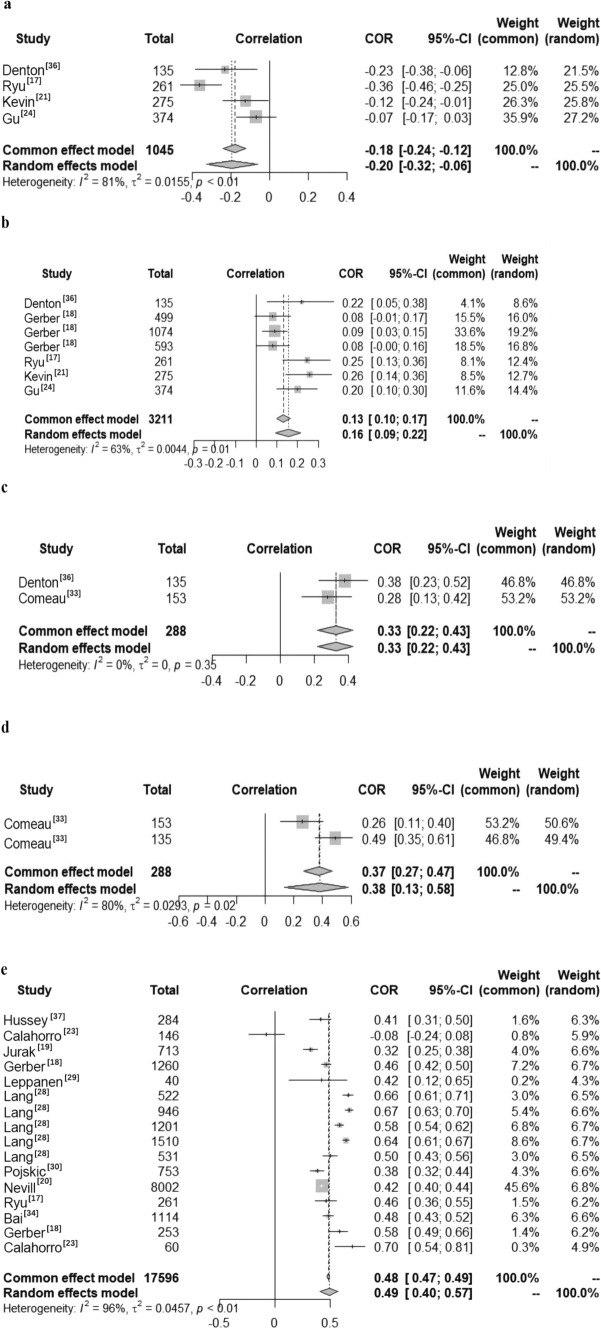
Forest diagram of the correlation between daily behavior (DB) and cardiorespiratory fitness (CRF) in children and adolescents. **a** Light physical activity (LPA) and CRF; **b** moderate to vigorous physical activity (MVPA) and CRF; **c** vigorous physical activity (VPA) and CRF; **d** high physical activity (HPA) and CRF; **e** total physical activity (TPA) and CRF. *COR* correlation

The included studies indicated a significant positive correlation between moderate to vigorous physical activity (MVPA) represented DB and CRF in children and adolescents aged 5–18 years (Fig. 3). A total of 21 studies reported data on the relationship between DB and CRF in children and adolescents. Among them, four studies reported the relationship between light physical activity (LPA) and CRF (Fig. 6a), seven reported the relationship between MVPA and CRF (Fig. 6b), two reported the relationship between vigorous physical activity (VPA) (Fig. 6c) and CRF, two reported the relationship between high physical activity (HPA) (Fig. 6d)and CRF, and 16 reported the relationship between total physical activity (TPA) and CRF (Fig. 6e). Heterogeneity testing revealed significant heterogeneity for LPA (*I*^2^ > 50%,* P* < 0.01), MVPA (*I*^2^ > 50%, *P* < 0.05), HPA (*I*^2^ > 50%, *P* < 0.05), and overall PA (*I*^2^ > 50%, *P* < 0.01); therefore, the random effects model was chosen for the meta-analysis. However, VPA studies showed no significant statistical heterogeneity (*I*^2^ < 50%, *P* > 0.05), and a fixed effects model was therefore chosen for meta-analysis.

The meta-analysis results showed that LPA was significantly negatively correlated with CRF among children and adolescents (COR = − 0.20, 95% CI − 0.32, − 0.06). MVPA, VPA, HPA, and overall PA were all significantly positively correlated with CRF (COR = 0.16, 95% CI 0.09, 0.22; COR = 0.33, 95% CI 0.22, 0.43; COR = 0.38, 95% CI 0.13, 0.58; COR = 0.49, 95% CI 0.40, 0.57).

#### Relationship Between Knowledge and Understanding (K&U) and CRF Among Children and Adolescents

**Fig. 7:**
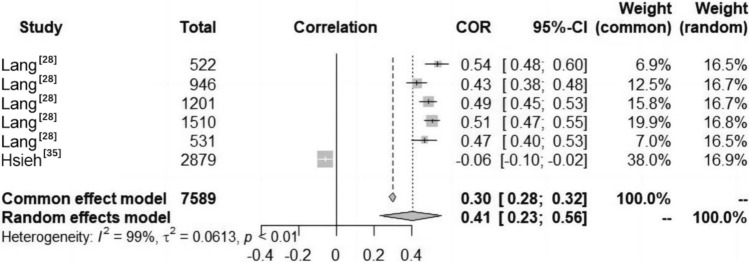
Forest diagram of the correlation between knowledge and understanding (K&U) and cardiorespiratory fitness (CRF) in children and adolescents. *COR* correlation

The included studies indicated a significant positive correlation between K&U and CRF in children and adolescents aged 5–12 years and a significant negative correlation between K&U and CRF in those aged 13–15 years; no correlation was observed in children and adolescents those aged 16–18 years (Fig. 3). The results were the same for both males and females across different studies. A total of six studies reported data on the relationship between K&U and CRF in children and adolescents (Fig. 7). Heterogeneity testing revealed significant heterogeneity among the studies (*I*^2^ > 50%, *P* < 0.01); thus, the random effects model was chosen for the meta-analysis. The meta-analysis revealed a positive correlation between K&U and CRF in children and adolescents (COR = 0.41, 95% CI 0.23, 0.56).

#### Relationship Between Motivation and Confidence (M&C) and CRF among Children and Adolescents

**Fig. 8:**
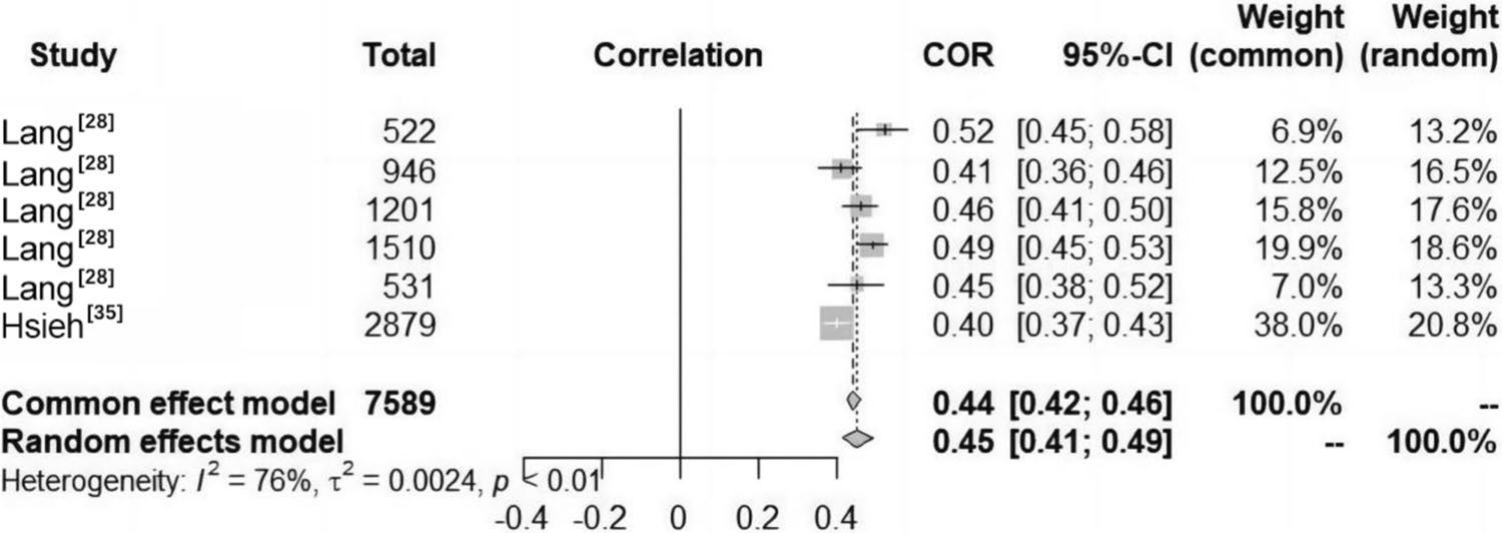
Forest diagram of the correlation between motivation and confidence (M&C) and cardiorespiratory fitness (CRF) in children and adolescents. *COR* correlation

The included studies indicated a significant positive correlation between M&C and CRF in children and adolescents aged 8–13 years (Fig. 3). No studies examined this relationship among other age groups. A total of six studies reported data on the relationship between M&C and CRF in children and adolescents (Fig. 8). Heterogeneity testing revealed significant heterogeneity among the studies (*I*^2^ > 50%, *P* < 0.01); thus, the random effects model was chosen for the meta-analysis. The meta-analysis revealed a positive correlation between M&C and CRF in children and adolescents (COR = 0.45, 95% CI 0.41, 0.49).

#### Sensitivity Analysis

The sensitivity analysis results showed that all studies fell within the 95% CIs for credibility, and there was no significant heterogeneity among the studies. The exclusion of any single study did not significantly affect the outcome measures, indicating that heterogeneity did not arise from a single study.

## Discussion Analysis

Research on the relationship between PL and CRF in children and adolescents is an emerging field, with the majority of related literature published after 2016 (85.7%) and a significant portion published after 2020 (42.9%). Comprehensive reviews of existing research progress and dynamics in this field have not been effectively conducted. Within the four dimensions of PL, preliminary review articles have attempted to explore the potential relationship between PC and DB and CRF, finding that DB characterized by active learning is closely associated with CRF in children and adolescents (aged 5–18 years) [38] and that PC in children (aged 8–13 years) is closely linked to CRF. However, systematic analyses and meta-analyses of the relationships between PL and CRF among children and adolescents are currently lacking. This study is the first known systematic review and meta-analysis of the relationship between PL and CRF among children and adolescents.

### Analysis of the Relationship Between PL and CRF Among Children and Adolescents

The systematic review and meta-analysis results showed that there was a significant positive correlation between overall PL and CRF in studies that included children and adolescents aged 8–13 years, regardless of sex. The combined effect results were statistically significant (COR = 0.64, 95% CI 0.58, 0.70). This result provides a new direction for exploring the factors influencing CRF and for developing intervention strategies. On the one hand, as a concept that governs contemporary sports reform and development [39], PL is an essential foundation for the comprehensive development of children and adolescents [40]. Its development and improvement have positive effects on the cardiorespiratory health of children and adolescents. On the other hand, interventions for improving CRF in children and adolescents should not be limited to only cardiovascular and respiratory functions but should focus on holistic development, encompassing various interrelated dimensions, including emotional, cognitive, and physical aspects [39]. Developing PL as an intervention direction can simultaneously improve both CRF and the overall physical and mental health of children and adolescents. However, considering the characteristics of the included studies, the age range of the subjects in the existing studies was limited to 8–13 years. More research evidence is needed for earlier and later stages of childhood and adolescence, which requires further research efforts.

### Analysis of the Relationship Between PC and CRF in Children and Adolescents

This systematic review and meta-analysis revealed a significant positive correlation between PC and CRF in male and female children and adolescents aged 7–14 years. The combined effect results were statistically significant (COR = 0.74, 95% CI 0.69, 0.79), suggesting that PC is an effective factor for promoting CRF in children and adolescents. This finding is consistent with the findings of a systematic review and meta-analysis published by Comeras et al. in 2021 [41]. That review indicated that PC is a potential factor influencing CRF in children and adolescents [41]. A systematic review published by Cattuzzo et al. in 2016 revealed a significant positive correlation between PC and CRF among children and adolescents [42]. Concurrently, it was found that children with lower PC levels have poorer physical health than those with higher PC levels [43], indicating that PC can effectively predict the CRF levels of children and adolescents [44–47]. The association between PC and CRF suggests that interventions to improve CRF could incorporate the development of fundamental motor skills (e.g., jumping, throwing). These PC interventions might have direct or indirect effects that can further enhance the CRF levels of children and adolescents. However, whether PC has the same promoting effect on CRF in preschool children requires further investigation.

### Analysis of the Relationship Between DB and CRF Among Children and Adolescents

This systematic review and meta-analysis revealed a significant positive correlation between MVPA-based DB and CRF in studies that included male and female children and adolescents aged 5–18 years. The combined effect results were statistically significant (COR = 0.16, 95% CI 0.09, 0.22), indicating that MVPA is a potential factor for improving CRF in children and adolescents. However, there was a significant negative correlation between LPA and CRF (COR = − 0.20, 95% CI − 0.32, 0.06). VPA, HPA, and overall PA were all significantly positively correlated with CRF (COR = 0.33, 95% CI 0.22, 0.43; COR = 0.38, 95% CI 0.13, 0.58; COR = 0.49, 95% CI 0.40, 0.57). The reasons for this might be that not all children and adolescents can achieve and maintain medium- to high-intensity levels of physical activity [48]. Schools provide an ideal environment for participating in physical activities [49], but the school environment might also restrict the intensity of activities needed to improve CRF [50]. In recent years, many researchers have explored the relationship between different intensities of physical activity and CRF in children and adolescents [32, 36, 37], yielding fruitful results. Systematic reviews and meta-analyses have also investigated the correlation between active video gaming and CRF in children and adolescents, suggesting that active video gaming can significantly enhance CRF and musculoskeletal abilities [41]. In 2011, a review analyzed the relationship between a DB characterized by active learning and CRF in children and adolescents and demonstrated a positive correlation between active learning behavior and adolescent CRF [38]. Recent studies suggest that engaging in longer periods of moderate to vigorous physical activity can lead to higher CRF levels in children and adolescents [51, 52].

### Analysis of the Relationship Between K&U and CRF Among Children and Adolescents

This systematic review and meta-analysis revealed a significant positive correlation between K&U and CRF in male and female children and adolescents aged 5–12 years. There was a negative correlation between K&U and CRF in those aged 13–15 years, but no correlation was found in those aged 16–18 years. The combined effect results were statistically significant among children and adolescents aged 5–12 years (COR = 0.41, 95% CI 0.23, 0.56), suggesting that K&U is an effective factor for promoting CRF in children and adolescents aged 5–12 years. This finding is consistent with the results of the systematic review and meta-analysis by Alves et al. in 2021, which showed a significant negative correlation between mental health issues and CRF in children and adolescents [53]. Moreover, higher CRF levels in the adolescent population are associated with better mental health, indicating that K&U can predict CRF in children and adolescents [54]. Studies have shown that the relationship between K&U and CRF in children and adolescents may be directly or indirectly linked to their impact on social relationships [55]. Thus, further empirical research is needed to provide a theoretical basis for better understanding the link between K&U and CRF in children and adolescents.

### Analysis of the Relationship Between M&C and CRF in Children and Adolescents

This systematic review and meta-analysis revealed a significant positive correlation between M&C and CRF in male and female children and adolescents aged 8–13 years. The combined effect results were statistically significant (COR = 0.45, 95% CI 0.41–0.49), indicating that M&C is an effective factor for promoting CRF in children and adolescents. This could be because M&C can promote engagement of children and adolescents in physical activities and socialization with peers, thereby increasing physical activity levels and reducing sedentary behavior, ultimately leading to improved CRF. Therefore, when designing intervention measures, efforts should be made to enhance children’s and adolescents’ confidence in participating in exercise and physical activities, which can help improve CRF levels.

### Limitations

Although this review strictly followed the PRISMA guidelines, there are still certain limitations: (1) The included studies were all observational studies, lacking experimental research outcomes. This may affect the strength of the causal relationship between PL and CRF. (2) There is high heterogeneity among the various studies, which could be due to the broad geographic distribution of the study subjects (from 23 countries or regions) and the variations in measurement tools for relevant indicators. In addressing this issue, this study used the REM instead of the CEM model and conducted sensitivity analyses to mitigate the impact of heterogeneity on the results.

## Conclusion

### Summary

1. There is a positive correlation between children and adolescents’ overall PL and CRF. This suggests that there is a high correlation between the development of physical literacy and the cardiorespiratory health of children and adolescents.
2. Regarding the dimensions of PC, DB, K&U, and M&C, all are positively correlated with CRF, and the results of male and female samples from the included studies are the same. This indicates that the health effects of developing physical literacy on cardiorespiratory health in children and adolescents are not restricted by dimensions or sex.
3. Within the DB dimension, TPA is significantly positively correlated with CRF. Specifically, MVPA, VPA, and HPA are all significantly positively correlated with CRF, whereas LPA is significantly negatively correlated with CRF. This suggests that daily behavior is highly correlated with cardiorespiratory fitness, and that the intensity of physical activity should be at least moderate to achieve this effect.

### Prospects

#### Conducting Relevant Experimental Studies

Based on the characteristics of the included literature, research on the relationship between PL and CRF among children and adolescents is still an emerging field and is in its early stages. Many of the included studies were published from 2016 to the present, and mostly from 2020 to the present. All included studies were observational studies, and the transition to experimental research has not yet occurred. Although observational studies can confirm the close relationship between PL and CRF, they cannot effectively explain the cause-and effect relationship between the two; thus, there is a need for well-designed experimental studies to supplement the evidence. Future research could design scientifically sound randomized controlled and crossover experiments focusing on PL and its various dimensions to explore its roles and mechanisms in influencing the CRF of children and adolescents, providing stronger scientific evidence for the establishment and improvement of CRF warning indicators and intervention strategies.

#### Investigating the Interaction Between Different Dimensions of Physical Literacy

This study confirmed that each dimension of PL is significantly positively correlated with CRF, indicating that improving any dimension of PL can have positive effects on improving the cardiorespiratory health of children and adolescents. However, existing research shows that there are close relationships between the various dimensions of PL. For example, DB can effectively increase PC levels in children and adolescents, and PC can increase DB and K&U levels [56–58]. Therefore, one dimension might exert interaction effects on another or multiple dimensions, enhancing or weakening their health effects on CRF in children and adolescents. Thus, investigating the interaction between the different dimensions of PL remains an important research topic. Future research could explore the interaction effects of the various dimensions of PL on the CRF of children and adolescents through data mining or multifactor combination intervention designs, enriching and improving the research framework of the relationship between PL and CRF among children and adolescents.

#### Conducting Research on Pre-school Children

Based on the preliminary literature search, the age range of research samples in the early stages of life is limited to children and adolescents, with the youngest sample being 5.5 years old [29], and there is no research available on the age group of 3–5 years, which consists of preschool children. Currently, investigators worldwide have developed evaluation indicator systems for the assessment of preschool children’s PL [21], and local investigators and teams have begun to develop measurement tools suitable for evaluating PL in preschool children in China [59]. With this foundation, the conditions for researching preschool children are gradually maturing, and future researchers, including the authors of this study, will strive to explore this population, providing evidence for the relationship between PL and CRF at an even earlier stage of human life.

## Declarations

### Data Availability

Data sets generated during the current review are available from the authors on reasonable request.
